# Preparation and Characterization of Thin-Film Solar Cells with Ag/C_60_/MAPbI_3_/CZTSe/Mo/FTO Multilayered Structures

**DOI:** 10.3390/molecules26123516

**Published:** 2021-06-09

**Authors:** Tsung-Wen Chang, Chzu-Chiang Tseng, Dave W. Chen, Gwomei Wu, Chia-Ling Yang, Lung-Chien Chen

**Affiliations:** 1 Department of Electronic Engineering, Institute of Electro-Optical Engineering, Chang Gung University, Chang Gung Memorial Hospital, Taoyuan 333, Taiwan; overcomer1984@gmail.com (T.-W.C.); D0427101@cgu.edu.tw (C.-C.T.); mr5181@cgmh.org.tw (D.W.C.); 2 Department of Power Mechanical Engineering, National Tsing Hua University, Hsinchu 300, Taiwan; s9833815@m98.nthu.edu.tw; 3 Department of Electro-Optical Engineering, National Taipei University of Technology, Taipei 106, Taiwan; Ocean@ntut.edu.tw

**Keywords:** thin-film solar cell, C_60_, perovskite, CZTSe

## Abstract

New solar cells with Ag/C_60_/MAPbI_3_/Cu_2_ZnSnSe_4_ (CZTSe)/Mo/FTO multilayered structures on glass substrates have been prepared and investigated in this study. The electron-transport layer, active photovoltaic layer, and hole-transport layer were made of C_60_, CH_3_NH_3_PbI_3_ (MAPbI_3_) perovskite, and CZTSe, respectively. The CZTSe hole-transport layers were deposited by magnetic sputtering, with the various thermal annealing temperatures at 300 °C, 400 °C, and 500 °C, and the film thickness was also varied at 50~300 nm The active photovoltaic MAPbI_3_ films were prepared using a two-step spin-coating method on the CZTSe hole-transport layers. It has been revealed that the crystalline structure and domain size of the MAPbI_3_ perovskite films could be substantially improved. Finally, n-type C_60_ was vacuum-evaporated to be the electronic transport layer. The 50 nm C_60_ thin film, in conjunction with 100 nm Ag electrode layer, provided adequate electron current transport in the multilayered structures. The solar cell current density–voltage characteristics were evaluated and compared with the thin-film microstructures. The photo-electronic power-conversion efficiency could be improved to 14.2% when the annealing temperature was 500 °C and the film thickness was 200 nm. The thin-film solar cell characteristics of open-circuit voltage, short-circuit current density, fill factor, series-resistance, and Pmax were found to be 1.07 V, 19.69 mA/cm^2^, 67.39%, 18.5 Ω and 1.42 mW, respectively.

## 1. Introduction

More than 80% of the world’s current energy demands are satisfied by exhaustible fossil fuels. It is already having significant impacts on human health and the global environment. The world has been on the verge of irreversible climate change for years. Moreover, global energy demands have been predicted to be doubled by the year 2050 [1]. The quest for a realistic solution is on the priority list for the research community. On the other hand, organic–inorganic hybrid materials, particularly the perovskite family, have shown great potential for industrial applications in field-effect transistors, solar cells, light-emitting diodes, biochemical sensors, and photon detectors [2,3,4]. 

In the last decade, the power-conversion efficiency (PCE) of lead halide perovskite (CH_3_NH_3_PbX_3_, X = Cl, Br or I)-based thin film photovoltaic devices has been improved dramatically [5,6,7,8]. Organic metal halide materials have shown excellent properties for photon harvesting for solar cells, such as a low band gap and a large excitation diffusion length [9]. The optical band gap of organic metal perovskite (CH_3_NH_3_PbI_3_; MAPbI_3_) is about 1.5 eV. Therefore, this material favors efficient carrier generation and transport to electrodes. It can harvest sunlight radiation from the ultraviolet (300 nm) to the infrared region (800 nm) by adequately adjusting the composition of the three-dimensional perovskite.

Stranks et al. reported a large range length of excitation diffusion greater than 1 micrometer in the mixed halide perovskite [10] Miyasaka et al. studied organo-metal halide perovskite solar cells with a mesoporous TiO_2_ structure. In 2009, they used CH_3_NH_3_PbI_3_ and CH_3_NH_3_PbBr_3_ nano-crystals as the active absorbing materials, and achieved an efficiency of 3.8% [11]. In 2012, Kim et al. developed fully solid-state perovskite solar cells with a fluorine-doped tin oxide (FTO)/mesoporous TiO_2_/perovskite/2,2′,7,7′-Tetrakis(*N*,*N*-diphenylamino)-9,9-spirobifluorene (Spi- roOMeTAD)/gold structure, achieving an efficiency greater than 9% [12]. 

Jeng et al. investigated the first inverted planar structure of a perovskite solar cell, and a similar device structure was used in an organic solar cell [13]. This inverted structure was typically represented as indium tin oxide (ITO)/poly (3,4-ethylenedioxythiophene):poly (styrene sulfonate)(PEDOT:PSS)/perovskite/(6,6)-phenyl-C61-butyric acid methylester (PCBM)/silver. The PEDOT:PSS has been generally used as a hole-transport material (HTM). The conventional and inverted structures could be distinguished by the directions of the transport of carriers. However, the traditional hole-transport material of PEDOT:PSS was somewhat acidic. It could corrode the ITO and reduced the stability of the devices [14,15]. In practice, FTO has been developed to be used to replace ITO as the transparent conducting oxide (TCO) when a post-annealing process in air is required for the thin film structures. FTO could be deposited on top of an optical glass-substrate, such as in dye-sensitized solar cells or perovskite-based solar cells, where a TiO_2_ layer is needed on top of the TCO and will require thermal annealing treatment. The ITO’s electrical properties can be degraded in the presence of oxygen at relatively high temperatures, such as around 500 °C. On the other hand, FTO is much more stable in such high annealing temperature conditions. 

Metal–selenide materials have received considerable attention owing to their excellent optical characteristics and electronic properties. These materials are very attractive for applications in quantum dots [16,17,18,19], photovoltaic devices and gas sensors [20,21,22]. Todorov et al. investigated CZT(S,Se) as an absorber layer in Kesterite solar cells. This lower-cost and earth-abundant material exhibited a power conversion efficiency above 11% [23]. In addition, Cd-free kesterite-based Cu2ZnSnSe4 (CZTSe)/In2S3 could be fabricated by chemical spray pyrolysis [24]. An aqueous solution has also been developed as the HTM layer in halide photovoltaic solar cells [25]. Cu_2_ZnSn(S_x_Se_1-x_)_4_ has emerged as a promising candidate for scalable photovoltaic applications, and its band gap ranges between 1.0 eV and 1.5 eV, depending on the value of x [26,27]. However, few investigations of metal–selenide as a carrier-transport material in perovskite solar cells have been reported [28,29]. In addition, fullerene C_60_ is an exciting all-carbon molecule [30]. Wojciechowski et al. studied an efficient perovskite solar cell by using it as an n-type compact layer for normal n-i-p structures. They suggested that the solar cells with C_60_ carrier charge selective layers could preserve more photocurrent due to increased electronic coupling. It also decreased the non-radiative decline on contact under forward bias [31]. Warner et al. also evaluated the nanostructure in copper phthalocyanine-C_60_ solar cell blends. Their solar cell’s optimal efficiency could be improved by an alternative film growth procedure [32,33,34]. In this study, CH_3_NH_3_PbI_3_ perovskite solar cells have been prepared using CZTSe as a hole-transport layer. C_60_, an n-type semiconductor material, was vacuum evaporated to be the electronic; transport layer (ETL). The solar cells’ photo-electronic characteristics were investigated using a Keithley 2420 programmable source meter system under an irradiation power density of 1000 W/m^2^. The purpose of this study has been to understand the effects of thin film thicknesses and energy level alignments in multilayered structures. The processing parameters should also play important role in improving the design of next generation perovskite solar cells. A successful development in the field could help to pave the way for new thin-film solar cells to contribute to at least part of the energy demand. This would indeed be beneficial to the global environment.

## 2. Results and Discussion

**Figure 1:**
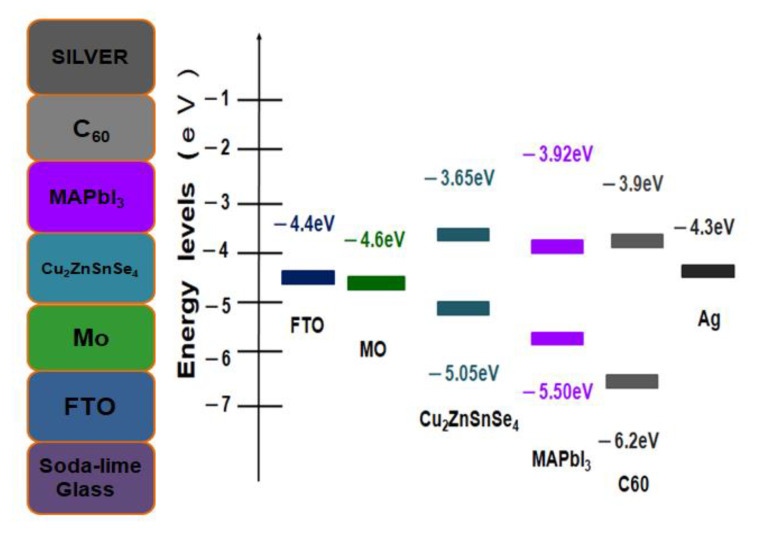
The energy level diagrams of Ag/C_60_/MAPbI_3_/CZTSe/Mo/FTO multilayered thin-film solar cell on glass substrate.

Figure 1 shows the corresponding energy levels of the Ag/C_60_/CH_3_NH_3_PbI_3_ (MAPbI_3_) perovskite/Cu_2_ZnSnSe_4_ (CZTSe)/Mo/FTO multilayered photovoltaic structures [35,36,37] on glass substrates that have been investigated in this study. The proposed cell structure can be improved by varying different material layer properties, such as thickness, carrier concentration, and defect density. It has been noted that holes may travel more favorably than electrons. The device performances are also highly influenced by the different thin film stacks, as well as the electronic properties at the interfaces. Nevertheless, the energy level diagrams indeed suggested a true heterojunction planar thin-film solar cell.

**Figure 2:**
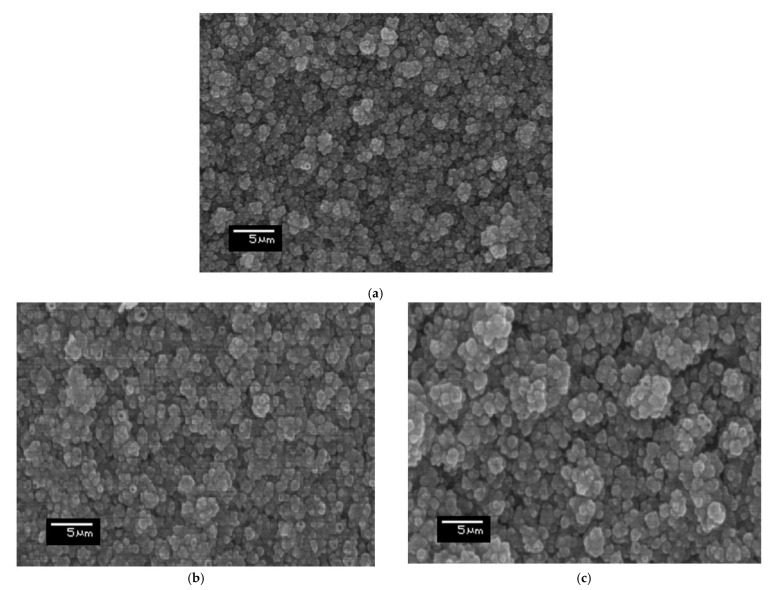
Top-view SEM micrographs of the sputtered CZTSe films that were thermally annealed at (**a**) 300 °C, (**b**) 400 °C, and (**c**) 500 °C.

The top-view SEM micrographs of the CZTSe films on Mo/FTO glass substrates, following the thermal annealing process at the various temperatures, are displayed in Figure 2. It has been clearly shown that the grain size increased with the increasing annealing temperature. A crystallized and dense CZTSe film was observed when the annealing temperature was exceeding 300 °C. However, in Figure 2a, the CZTSe thin film surface, annealed at 300 °C, has appeared with relatively smaller crystalline grains. Some defect pinholes can be clearly observed. The defect phenomena were likely caused by the incomplete sintering of the film material. On the other hand, Figure 2b shows larger crystalline grains with less defect pinholes for the CZTSe thin film that had been annealed at a higher temperature of 400 °C. In addition, after the highest 500 °C annealing temperature process for one hour (see Figure 2c), the thin film surface revealed the largest crystalline grains, with almost undiscernible defect pinholes at the same magnification scale.

**Figure 3:**
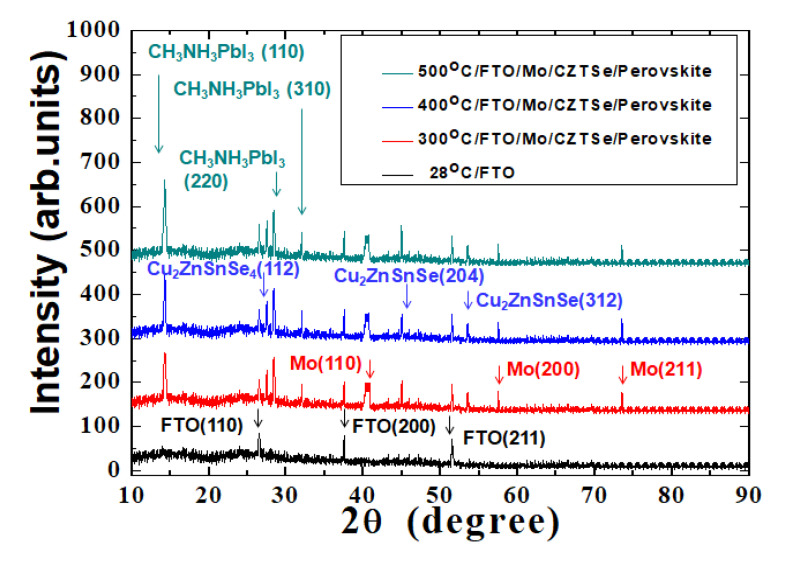
XRD spectra of MAPbI_3_/CZTSe/Mo/FTO multilayered thin-film structures after thermal annealing at various temperatures.

A lower annealing temperature would produce thin films with lots of defects or pinholes on the surface. It is known that high-quality perovskite film is critical for the fabrication of multilayered thin-film photovoltaic devices. The X-ray diffraction (XRD) patterns of the MAPbI_3_ perovskite films on CZTSe HTM films, following thermal annealing at the various temperatures, are examined in Figure 3. The spectra of the MAPbI_3_ perovskite films that were deposited on the CZTSe HTM films that were thermally annealed at 300 °C, 400 °C, and 500 °C all showed one dominant CH_3_NH_3_PbI_3_ (110) diffraction peak at 14.3°, indicating a strong preferential and prominent orientation in the crystal growth direction. When the annealing temperature was increased, the full-width at half-maximum (FWHM) of the CH_3_NH_3_PbI_3_ (110)diffraction peak was slightly improved. A smaller FWHM suggested a larger averaged domain size with improved perovskite crystallization in the multilayer structures of the thin-film solar cells. 

The function of the MAPbI_3_ perovskite film is as an active photovoltaic layer, and the CZTSe film provides the hole-transporting layer. The CZTSe thin film layer and the MAPbI_3_ thin film layer can be annealed and sintered altogether. Their energy band gaps are similar for trapping light. As a result, this trapping of light operates to form pairs of an excited electron and an associated electron-hole for subsequent recombination. It can affect the photo-electronic power conversion efficiency and further correlations between films and the solar cells’ multilayer structures. 

**Figure 4:**
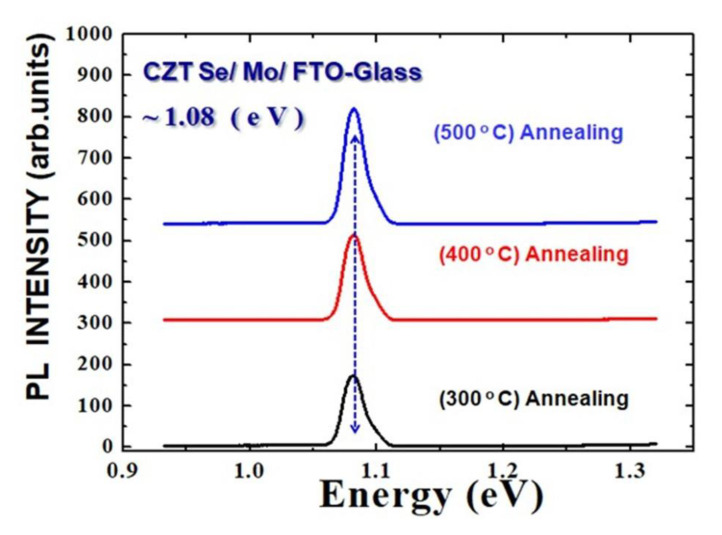
Room-temperature PL spectra of MAPbI_3_ deposited on CZTSe/Mo/FTO glass substrates following the annealing treatments at 300, 400, and 500 °C.

Figure 4 shows the room-temperature photoluminescence (PL) spectra of the MAPbI_3_ perovskite films that were deposited on CZTSe/Mo/FTO glass substrates following the annealing temperature at 300 °C, 400 °C, and 500 °C. The corresponding emission peak was clearly observed at ~1.08 eV. The photoluminescence was measured using fluorescence spectrophotometer equipment (Hitachi F-7000). The intensity of the PL spectrum peak is related to the lifetime of the exciton in the MAPbI_3_ perovskite film. The exciton appears when a mobile concentration of energy in a crystal is formed by an excited electron and an associated electron-hole. As the optical stimulation was intensified during the experiments, the excitons increased in number and the electron-hole pairs could cancel each other out. The current density–voltage (J–V) characteristics were evaluated using a Keithley 2420 programmable source meter under irradiation from a 1000 W xenon lamp. The system forward scan rate was 0.1 V/s. The irradiation power density on the surface of the solar cell sample was nominally calibrated to 100 mW/cm^2^. As the annealing temperature was increased from 300 °C to 500 °C, the PL intensity of the MAPbI_3_ perovskite film also become increased. This indicated that the decomposition rate of excitons at the interface between the MAPbI3 material and the CZTSe layer was quickly achieved. The carrier lifetime is generally limited by surface-related nonradiative recombination [38]. The MAPbI_3_/CZTSe films were grown by annealing, which resulted in ohmic contact formation, and the low contact resistance of the CZTSe film with the back electrode Mo film. The stability of MAPbI_3_ perovskite solar cells is related to not only selective contact materials, but also the MAPbI_3_ perovskite’s constitutional elements and morphology. In addition, MAPbI_3_ perovskite materials are sensitive to moisture and can be degraded rapidly. CZTSe thin film can be an ideal HTM candidate to be incorporated into the bulk MAPbI_3_ perovskite layer to improve the device’s stability [39].

**Figure 5:**
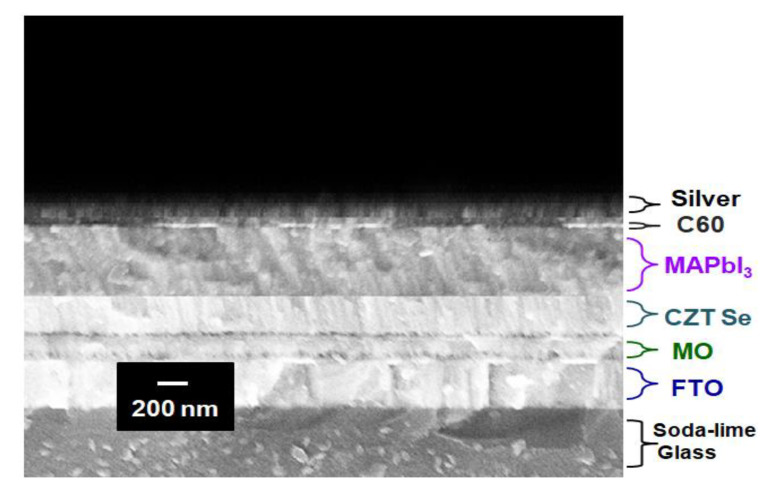
Cross-sectional SEM micrograph of Ag/C_60_/MAPbI_3_/CZTSe/Mo/FTO multilayered structures on glass substrate.

**Figure 6:**
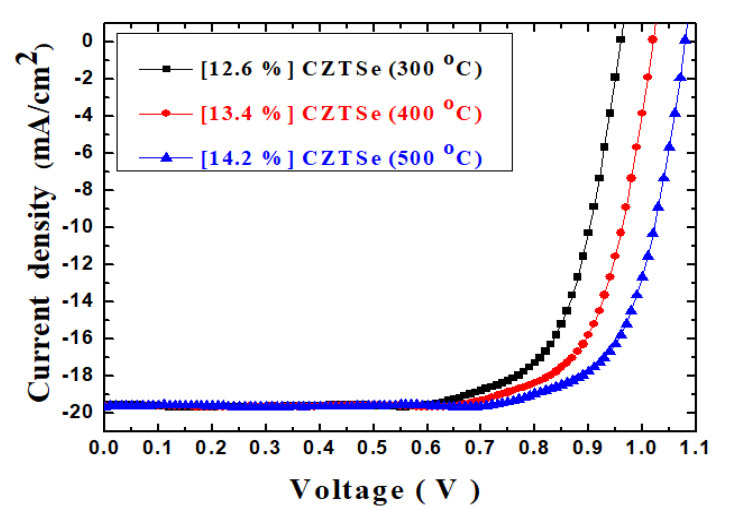
J–V curves for the Ag/C_60_/MAPbI_3_/CZTSe/Mo/FTO multilayered thin-film solar cells with various thermal annealing processes under 100 mW/cm^2^ illumination (AM 1.5G).

Figure 5 displays an example of the cross-sectional scanning electron micrograph (SEM) of the Ag/C_60_/MAPbI_3_/CZTSe/Mo/FTO multilayered thin-film structures on a glass substrate. This sample was annealed at 500 °C during its preparation. These thin films exhibit mainly smooth interfaces, free of pinholes, outgrowth orientations and micro cracks. Particulates originate from a target material via the mechanism of splashing [29]. In the experiments, it was found that the distribution density of particulates could be significantly reduced by the thermal annealing process. The 500 °C temperature treatment was sufficiently high to produce a clean, smooth surface. The SEM cross-sectional image clearly revealed a densely packed columnar structure. After all, Figure 6 illustrates the plotted current density–voltage (J–V) curves for the Ag/C_60_/MAPbI_3_/CZTSe/Mo/FTO multilayered thin-film solar cells with the various thermal annealing processes under 100 mW/cm^2^ illumination (AM 1.5G). In addition, the summarized photovoltaic characteristics of these thin-film solar cell devices are listed in Table 1. The photo-electronic power-conversion efficiency was improved from 12.6% to 14.2% as the annealing temperature was increased from 300 °C to 500 °C, mainly owing to the increase in the number of photon-generated carriers that were extracted and injected into the electrode because of the series resistance (Rs) in the device. The solar cell device exhibited a lower series resistance of 18.5 Ω when its HTM film was annealed at the higher temperature of 500 °C. This is depicted by the smoothness of the interface between the multilayered films of MAPbI_3_ and CZTSe, which resulted in good coverage. As already indicated in Table 1, the annealing at 300–500 °C reduced the series resistance of the devices. In addition, the open-circuit voltage (V_OC_) increased from 0.95 V to 1.07 V. The short-circuit current density (Isc) slightly improved from 19.57 mA/cm^2^ to 19.69 mA/cm^2^. Furthermore, the fill factor (FF) was good at 67–68%. The fill factor is defined as the ratio of the maximum electric output power Pmax of the solar cell to the product of the open-circuit voltage and the short-circuit current at the maximum electric power output. That is, it is the maximum power of the rectangle in the current-voltage characteristic plot. In fact, the fill factor is strongly affected by series resistance and shunt resistance. It is common to use only fill factor to simultaneously summarize these two effects. Any increase in series resistance or decrease in shunt resistance can reduce the fill factor, which in turn reduces the conversion efficiency. The equivalent circuit of an ideal solar cell can be mathematically modeled [40]. The photo-generated current density is simulated by a constant current source, which provides a constant current under light illumination. However, the FF cannot reach 100% due to the fact that the J–V curve can never be rectangular. It has been noted that most model equations for FF are somewhat empirical. The underlying factors may sometimes have a competitive relationship with each other. Their interactions determine how the current flows in the device. The FF also decreases with an increased cell area. Therefore, these factors often interact with each other in a complicated way. In practice, solar cell efficiency can be expressed by three important parameters: open-circuit voltage, short-circuit current, and fill factor. As a result, to improve the efficiency of solar cells, we must increase the open-circuit voltage and the short-circuit current, such as the photocurrent, a fill factor, in order to reduce series resistance and leakage current. The Pmax was found to be up to 1.42 mW. The improvement in photovoltaic performance may be attributed to the increase in the speed of the exciton decomposition, such that the electrons are separated and extracted quickly to the Mo/FTO glass substrates.

**Figure 7:**
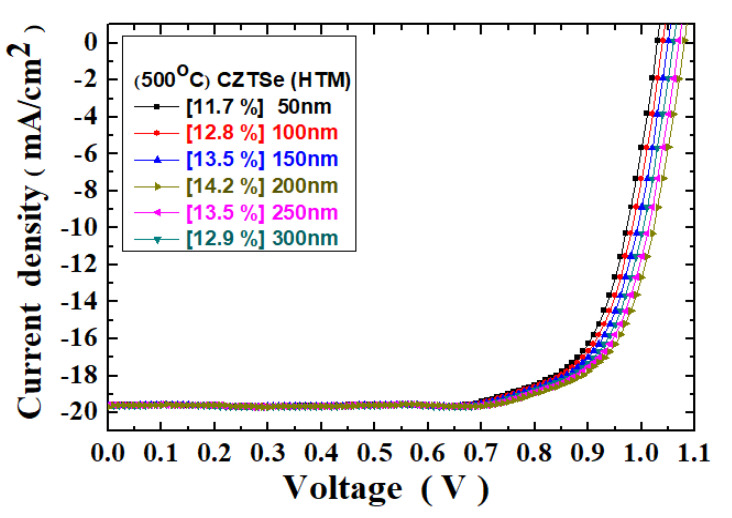
J–V curves for the Ag/C_60_/MAPbI_3_/CZTSe/Mo/FTO thin-film solar cells with the various CZTSe film thicknesses, also under 100 mW/cm^2^ illumination (AM 1.5G).

The multilayered structural parameters of the Ag/C_60_/MAPbI_3_/CZTSe/Mo/FTO thin-film solar cells were also investigated in the experiments. For example, Figure 7 shows the current density–voltage curves for the thin-film solar cells with the CZTSe HTM films of different thicknesses. All the CZTSe films were annealed at 500 °C, and the photovoltaic cell tests were measured under 100 mW/cm^2^ illumination (AM 1.5G). The solar cell photovoltaic characteristic parameters of the Ag/C_60_/MAPbI_3_/CZTSe/Mo/FTO thin-film solar cells are summarized in Table 2. The PCE value of the Ag/C_60_/MAPbI_3_/CZTSe/Mo/FTO solar cells was found to be improved from 11.7% to 14.2% as the thickness of the CZTSe HTM film increased from 50 nm to 200 nm. However, further increasing the thickness of the CZTSe HTM film to 300 nm would reduce the PCE to 12.9%, owing to the increased series resistance effect in the solar cell, as shown in Table 2.

**Figure 8:**
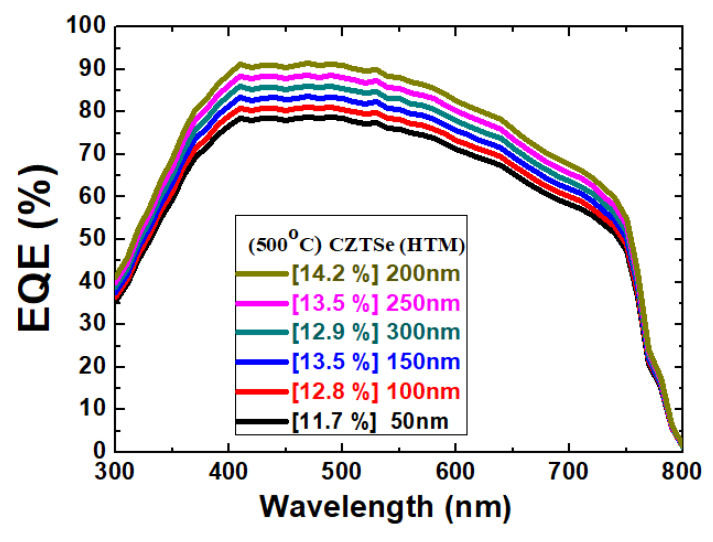
EQE spectra for the Ag/C_60_/MAPbI_3_/CZTSe/Mo/FTO thin-film solar cells with various CZTSe film thicknesses.

On the other hand, the fill factor of the thin-film solar cell was increased from 58.64% to 67.39% as the film thickness was increased from 50 nm to 200 nm. However, when the thickness further increased to 300 nm, the fill factor degraded to a lower value at 63.22%. The FF is an important parameter that determines the power conversion efficiency of a solar cell. As discussed earlier, there are several factors that can significantly influence FF. They even interact with each other. A more fundamental investigation can be further carried out to resolve the complicated relationship. In our measurement results, the series resistance value declined from 21.4 Ω to 18.5 Ω, while the HTM film thickness increased from 50 nm to 200 nm. Nevertheless, a further increase in film thickness to 300 nm increased the series resistance value to 20.6 Ω. These effects on the series resistance of the solar cells are detailed in Table 2. In addition, the open-circuit voltage has been shown to be in the range of 1.02–1.07 V. The short-circuit current density was maintained at 19.5–19.7 mA/cm^2^. The Pmax was estimated 1.17–1.42 mW. The external quantum efficiency (EQE) spectra at different thicknesses are presented in Figure 8. The improvement seems to be more profound than the change in the J_SC_ value. Nevertheless, the maximum EQE value was found in the 200 nm sample.

Any intrinsic characteristics of semiconductor materials inevitably involve more or less resistance. Therefore, series resistance will be generated in the solar cell. This is related to the junction depth, the impurity concentration of p-type, and the n-type in the region. On the other hand, any p-type or n-type’s electrode in a solar cell has another current that does not pass through the ideal p–n junction, which will cause a leakage of current. This may be due to the recombination of electron/electron-hole pairs in the depletion zone.

The internal recombination current or surface recombination current of the semiconductor material is an incomplete isolation of the elemental leakage current of the device. The leakage current then develops shunt-resistance due to the metal contact penetrating the p–n junction. However, the existence of the series resistance makes the short-circuit current relatively small, and the parallel resistance is not enough to reduce the open-circuit voltage. These two factors present the main causes for the decrease in the conversion efficiency of a solar cell. When the series resistance is increased, the slope of the straight line of the current–voltage curve becomes smaller. In addition, the parallel resistance is not large enough, and the slope of the straight line of the reverse direction becomes large. As to the fill factor, open-circuit voltage and short-circuit current, it can be drastically decreased, which in turn causes a reduction in the PCE conversion efficiency. According to the current density–voltage curves and the PCE values, the optimal thickness of the CZTSe HTM thin film was recommended to be 200 nm.

It has been noted that the photovoltaic stability is very important for the practical application of solar cells [25,41]. Long-term operation stability tests can be further carried out on solar cells and modules. These include indoor continuous irradiance tests, as well as outdoor irradiance tests, perhaps under simulated conditions. Additionally, an accelerated test can involve high temperatures and moisture. This is important, particularly for the perovskite solar cells. We plan to start by investigating the device’s components in encapsulated test structures. On the other hand, international standards for measuring photovoltaic modules should be followed, such as thermal cycling and humidity–freeze tests. A minimum loss in conversion efficiency is then highly desired.

## 3. Materials and Methods

In the experiments, molybdenum (Mo) metal thin-film was sputtered on FTO-coated glass substrates (Ruilong Glass) to be used as a back electrode contact layer [42]. A relatively high metal work function provides possible ohmic contact to the absorber layer. It decreases the barrier height for the charge carrier at the back contact interface, and in conjunction with a suitable surface recombination speed, can improve solar cell performance. The FTO glass substrates were subsequently cleaned using acetone (Merck), ethanol (Sigma-Aldrich), and deionized water, each for 10 min. Molybdenum is a kind of metal element material with chemical symbol Mo. Its atomic number is 42. It is a gray transition metal, used as a back electrode layer in this solar cell’s multilayer structures. The Mo thin film was prepared by RF magnetron sputtering using a Mo target (Ultimate Materials Technology) on commercially available FTO glass substrates. The Ar flow rate and radio-frequency (RF) power were maintained at 40 sccm and 80 W, respectively. The Mo thin film’s thickness was 200 nm approximately. Moreover, the CZTSe thin film layers were deposited by RF magnetron sputtering using a CZTSe target (Ultimate Materials Technology). This time, the Ar flow rate and RF power were maintained at 30 sccm and 60 W, respectively. The CZTSe film was sputtered on Mo metal thin film as a hole-transport layer in the multilayer structures [43].

The solution-processable MAPbI_3_ perovskites were prepared on CZTSe films to fabricate an inverted perovskite solar cell. The optical, structural, and surface properties of CZTSe films could then be examined. The relationship between the performance of solar cells and the properties of CZTSe films is useful in designing a photovoltaic device. The final CZTSe thin film had a thickness of ~200 nm, although several different thicknesses were also deposited for comparison. After the sputtering, the CZTSe thin films were annealed at 300 °C, 400 °C, or 500 °C in a tube furnace for one hour. The PbI_2_ (Alfa-Aesar) and MAI (Luminescence Technology) were dissolved in a co-solvent containing dimethyl sulfoxide (Emerald Scientific) and γ-butyrolactone (Sigma-Aldrich) (in volume ratio = 1:1) to constitute a perovskite precursor solution. The precursor solution was then coated onto the CZTSe films by a two-step spin-coating method at 1000 rpm and 5000 rpm for 10 and 20 s, respectively, in a glove box that was filled with highly pure nitrogen. The wet spinning thin film was quenched by dropping 50 µL of anhydrous toluene (Sigma Aldrich Corporation, St. Louis, MO, USA) at 17 s. After the spin coating process, the thin film of perovskite precursor solution was annealed at 100 °C for 10 min. The MAPbI_3_ perovskite thin film with a thickness of ~600 nm was thus deposited.

Furthermore, the C_60_ (fullerene) is an n-type semiconductor material. Here, its function was as an electronic transport layer in the solar cell’s multilayer structures. C_60_ powder (Uni-Onward Co., Pingzhen District, Taoyuan City, Taiwan) was prepared on a molybdenum boat in the vapor chamber of a vacuum evaporation system. It was deposited directly on the MAPbI_3_ perovskite films. The C_60_ thin film with a thickness of 50 nm was deposited approximately. C_60_ can increase the strength of metal materials by alloying, plastic deformation and heat treatment. Due to the existence of a three-dimensional highly delocalized electron conjugated structure in the C_60_ molecule, it has good optical and nonlinear optical properties [44,45]. In addition, C_60_ is an inexpensive and easily accessible material. The last electrode layer of silver (Ag) was deposited over the C_60_ n-type semiconductor material. A silver ingot (Gredmann Materials Technology) was prepared on a tungsten boat in the vapor chamber for evaporation. It was deposited above the C_60_ material. The silver thin film electrode had a thickness of about 100 nm. Additionally, the molybdenum/tungsten boats were connected to an external power supply and provided with a maximum current of 100 A and 90 A, respectively. The evaporation equipment model was PSE-1.5KVA. The power supply equipment mainly provided a direct current to the molybdenum boat or tungsten boat, separately. Due to the resistance effect, the temperature of the molybdenum or tungsten boats would be increased significantly. Subsequently, the C_60_ and the silver electrode were deposited at thicknesses of 50 and 100 nm, respectively, by the thermal vacuum evaporator equipment.

**Figure 9:**
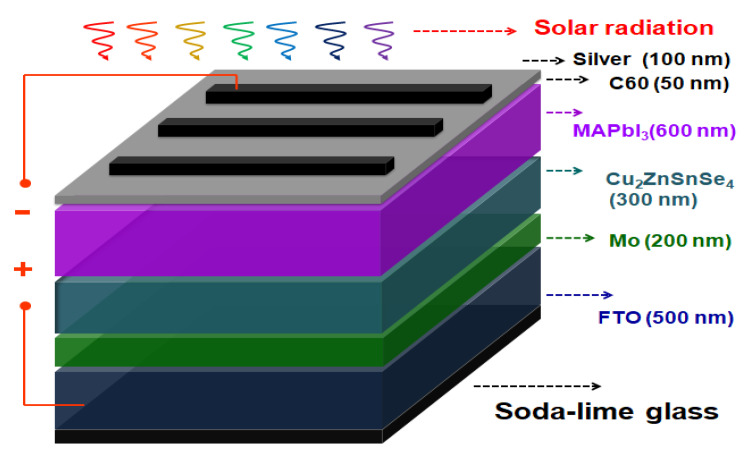
Schematic illustration of the multilayered structure of Ag/C_60_/MAPbI_3_/Cu_2_ZnSnSe_4_/Mo/FTO thin-film solar cell.

The Ag/C_60_/MAPbI_3_/CZTSe/Mo/FTO multilayered thin-film solar cell samples were further covered with a shadow mask to define an active area of 0.5 × 0.2 cm^2^ during the C_60_/silver deposition step. Thus, Figure 9 depicts a schematic illustration of the complete structure. The crystalline microstructures of the solar cell thin films were examined using a PANalytical X’Pert Pro DY2840 X-ray diffractometer (XRD) with Cu Kα radiation (λ = 0.154 nm). A field-emission scanning electron microscope (Zeiss Gemini SEM) was used to observe the surface morphology of the thin-film solar cells. The photoluminescence (PL) was measured using a fluorescence spectrophotometer (Hitachi F-7000, Tokyo, Japan). The current density–voltage (J–V) characteristics were evaluated using a Keithley 2420 programmable source meter under irradiation from a 1000 W xenon lamp. The equipment forward scan rate was set at 0.1 V/s. Finally, the irradiation power density on the surface of the solar cell sample was calibrated to 100 mW/cm^2^.

## 4. Conclusions

This paper presents the preparation, and investigations into the characteristics, of photovoltaic thin-film solar cells with Ag/C_60_/MAPbI_3_/CZTSe/Mo/FTO multilayer structures. The MAPbI_3_ perovskite films were deposited on CZTSe HTM films using a precursor solution made of PbI_2_ and MAI. The thermal annealing process improved the perovskite film in terms of its crystalline structure and domain size. This process ensured a smooth interface with good surface coverage in the multilayered thin-film structures. In combination with the designed film thicknesses and energy level alignments, it was efficient in reducing carrier recombination. Thus, it could result in rapid charge transition and low series resistance at the interface. From the experiments, we can conclude that the recommended solar cell device should have an active absorbing MAPbI_3_ perovskite film of 600 nm in thickness, a CZTSe HTM film of 200 nm in thickness, and a C_60_ ETL film of 50 nm in thickness, respectively. The thin-film multilayer structures have good omhic contact and minimal defects in their films. The thin-film solar cells were evaluated under 100 mW/cm^2^ illumination (AM 1.5G) for their current density–voltage curves. The photovoltaic characteristics exhibited improved the power-conversion efficiency from 12.6% to 14.2%. The open-circuit voltage was increased from 0.95 V to 1.07 V. The short-circuit current density was slightly improved from 19.57 mA/cm^2^ to 19.69 mA/cm^2^. The solar cell maintained a good fill factor at 67–68%. The device’s series resistance value was also reduced to 18.5 Ω. Furthermore, the maximum electric output power Pmax of the solar cell was 1.42 mW. We improved the MAPbI_3_ perovskite nanostructured photovoltaics using CZTSe HTM. The CZTSe films were deposited by one-step magnetron sputtering and annealed at different temperatures. It has been confirmed that the CZTSe film can be used as an HTM with C_60_ ETL to enhance the photovoltaic characteristic parameters.

## Figures and Tables

**Table 1:** Photovoltaic characteristics of the Ag/C_60_/MAPbI_3_/CZTSe/Mo/FTO solar cells following the CZTSe thermal annealing treatment at the various temperatures.

Annealing Temperature. (°C)	Voc (V)	Jsc (mA/cm^2^)	FF (%)	Eff (%)	Rs (Ω)	Pmax (mW)
300	0.95	19.57	67.77	12.6	20.1	1.26
400	1.01	19.63	67.58	13.4	19.3	1.34
500	1.07	19.69	67.39	14.2	18.5	1.42

**Table 2:** Photovoltaic characteristics of the Ag/C_60_/MAPbI_3_/CZTSe/Mo/FTO solar cells with various CZTSe film thicknesses, all thermally annealed at 500 °C.

CZTSe (HTM) Thickness (nm)	Voc (V)	Jsc (mA/cm^2^)	FF (%)	Eff (%)	Rs (Ω)	Pmax (mW)
50	1.02	19.56	58.64	11.7	21.4	1.17
100	1.03	19.59	63.43	12.8	20.2	1.28
150	1.05	19.61	65.56	13.5	19.8	1.35
200	1.07	19.69	67.39	14.2	18.5	1.42
250	1.05	19.62	65.53	13.5	19.6	1.35
300	1.04	19.60	63.22	12.9	20.6	1.29

## Data Availability

Not applicable.

## Sample Availability

Samples not available.
